# Serum and urinary biomarkers of collagen type‐I turnover predict prognosis in patients with heart failure

**DOI:** 10.1002/ctm2.267

**Published:** 2021-01-12

**Authors:** Tianlin He, Jesus D. Melgarejo, Andrew L. Clark, Yu‐Ling Yu, Lutgarde Thijs, Javier Díez, Begoña López, Arantxa González, John G. Cleland, Joost P. Schanstra, Antonia Vlahou, Agnieszka Latosinska, Harald Mischak, Jan A. Staessen, Zhen‐Yu Zhang, Vera Jankowski

**Affiliations:** ^1^ Mosaiques Diagnostics GmbH Hannover Germany; ^2^ Institute for Molecular Cardiovascular Research (IMCAR) RWTH Aachen University Aachen Germany; ^3^ Studies Coordinating Centre, Research Unit Hypertension and Cardiovascular Epidemiology KU Leuven Department of Cardiovascular Sciences University of Leuven Leuven Belgium; ^4^ Department of Academic Cardiology Castle Hill Hospital, University of Hull Cottingham UK; ^5^ Program of Cardiovascular Diseases, Centre for Applied Medical University of Navarra and IdisNA, Pamplona Spain; ^6^ CIBERCV Carlos III Institute of Health Madrid Spain; ^7^ Departments of Cardiology and Cardiac Surgery and of Nephrology University of Navarra Clinic Pamplona Spain; ^8^ Robertson Centre for Biostatistics and Clinical Trials, Institute of Health and Wellbeing University of Glasgow Glasgow UK; ^9^ Institute of Cardiovascular and Metabolic Disease INSERM Toulouse France; ^10^ Université Toulouse III Paul‐Sabatier Toulouse France; ^11^ Biotechnology Laboratory, Centre of Basic Research Biomedical Research Foundation of the Academy of Athens Athens Greece; ^12^ BHF Glasgow Cardiovascular Research Centre University of Glasgow Glasgow UK; ^13^ NPA Alliance for the Promotion of Preventive Medicine (APPREMED) Mechelen Belgium

Dear Editor,

Previous studies indicated an association of biomarkers reflecting collagen turnover with heart failure.^1^, ^2^ However, their value in predicting unfavorable outcomes is unknown. In the myocardium, the collagen matrix forms a scaffold that maintains the anatomical integrity and function of the heart. Collagen I is dominant among the four primary types of cardiac collagen.^3^ The abundance of collagen is the net result of the balance between synthesis and degradation.^4^, ^5^ Collagen degradation releases fragments to the circulation and urine. Here, we investigate if specific changes in collagen I turnover, detected based on urinary peptide fragments and circulating biomarkers, are associated with heart failure and subsequent clinical events.

Our study includes all 354 participants with suspected heart failure from the HOMAGE‐HULL subcohort^6^, ^7^ recruited between 2010 and 2014, whose baseline serum and urine samples were available. Table S1 shows their demographic and clinical data. We used capillary electrophoresis coupled with mass spectrometry (CE‐MS) to quantify type I alpha‐1 (COL1A1) fragments in urine, confining the analyses to 293 fragments present in at least 30% of subjects. For serum samples, we used ELISA Kit to quantify the collagen synthesis biomarkers carboxy‐terminal propeptide of procollagen type I (PICP),^8^ collagen degradation biomarkers carboxy‐terminal telopeptide of collagen type I (CITP),^9^ and matrix metalloproteinase (MMP‐1)^10^, which are known as good indicators of collagen turnover (Figure S1). The primary outcome is heart failure death. The rank‐normalized biomarker abundance was regressed with the time of survival in a Cox proportional hazard model corrected for six confounders: age, sex, body mass index, history of ischemic disease, heart rate, and serum creatinine. Significant biomarkers were combined into a classifier, using the support vector machine‐based MosaCluster software.^2^ Cox regression was used to determine if prognosis via the classifier was independent of the six confounders plus NT‐proBNP. All analyses were performed using Python 3.7 and were considered significant if *P* < .05.

There were 33 heart failure deaths during a median follow‐up of 6.4 years (interquartile range 4.9‐7.0 years). Figure 1 shows the −log10(P) plot of heart failure death with the 293 urinary COL1A1 fragments, in which eight were significant. Three of them were associated with an increased risk of heart failure death, all close to either the N‐ or C‐ terminus of mature collagen I. Five were associated with a decreased risk of heart failure death, all located in the central part of COL1A1. Table S2 presents the relative risk associated with a unitary change in fragment abundances and start amino acid positions on COL1A1 of the fragments. In serum, we detected a significantly positive association between heart failure death and CITP (HR 1.74, 95% CI 1.09‐2.78, *P* = .019) and MMP‐1 (HR 1.43, 95% CI 1.00‐2.04; *P* = .048), but not PICP (HR 0.99, 95%CI 0.69‐1.41; *P* = .96). Figure S2 displays the correlation between the eight urinary biomarkers and the three serum biomarkers. (HR = hazard ratio, CI = confidence interval of the hazard ratio)

**FIGURE 1:**
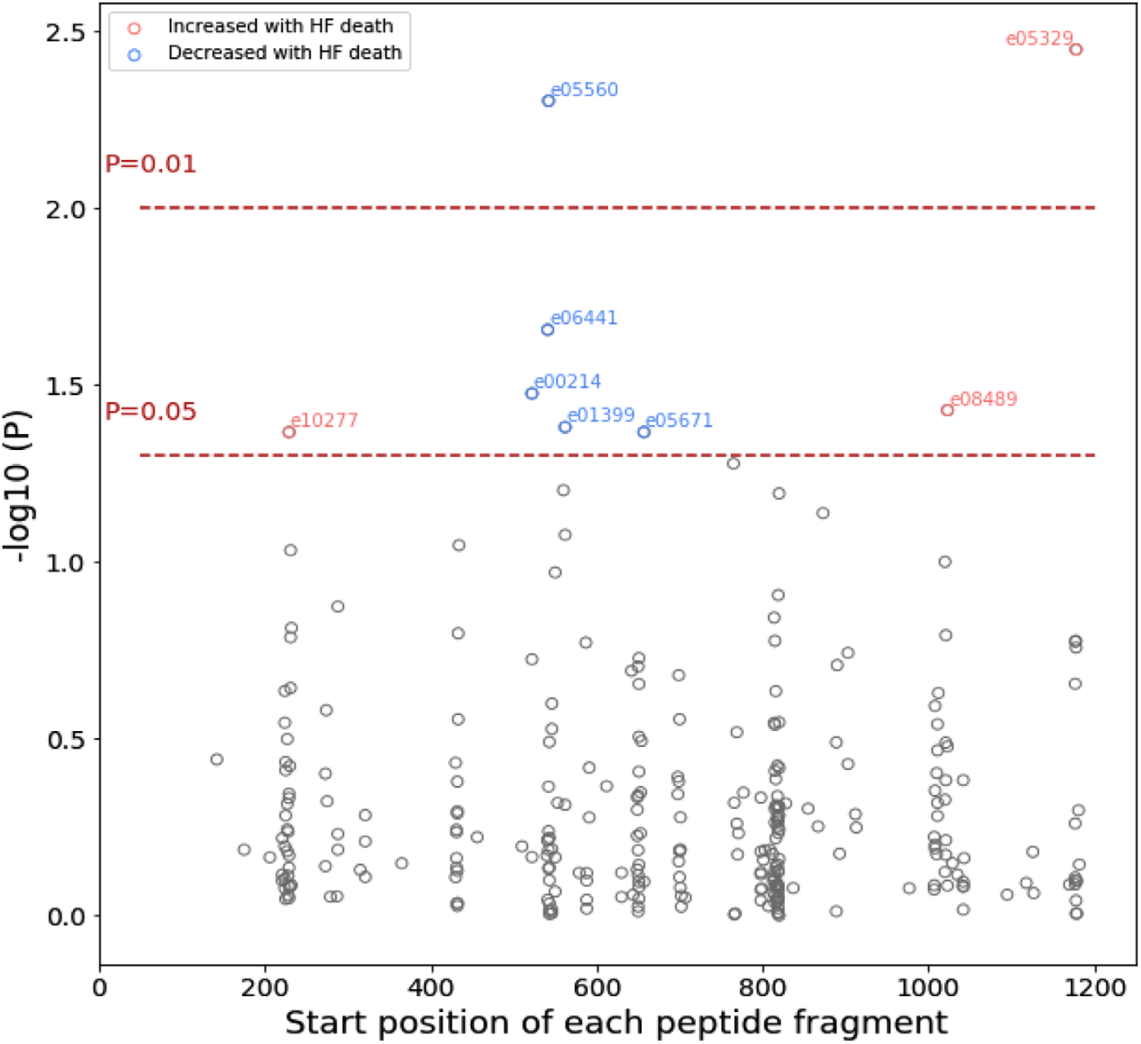
Log10(p) probability of urinary COL1A1 fragments associated with heart failure death in the multivariable Cox regression model in 354 patients. There are 33 heart failure deaths. The model was adjusted for age, sex, body mass index, history of ischemic disease, heart rate, and serum creatinine. The fragments were allocated according to their start position on COL1A1. The two horizontal lines denote the significance thresholds at *P* = .01 and *P* = .05, respectively. Only significant fragments (*P* < .05) were colored and annotated. Fragments that were increased in heart failure deaths were colored in red, otherwise in blue

These biomarkers were combined into a “urinary” and a “serum” classifier, respectively, whose components are listed in Table S3. We first verified that the association between the two classifiers and adverse outcome was independent of the other, and neither classifier was associated with noncardiovascular death (Table S4). There was no overlap between fragments associated with heart failure death and those associated with noncardiovascular death (Figure S3). We stratified the patients into quartiles by classifier scores. In analyses adjusted for the six cofounders and NT‐proBNP, increasing classifier score was associated with a higher incidence of heart failure death (Figure 2). The incidence of heart failure death in the highest quartile was eightfold higher than that in the lowest quartile (20.3% vs 2.53%, *P* < .001) for the urinary classifier, and fourfold higher for the serum classifier (14.0% vs 3.44%, *P* = .022).

**FIGURE 2:**
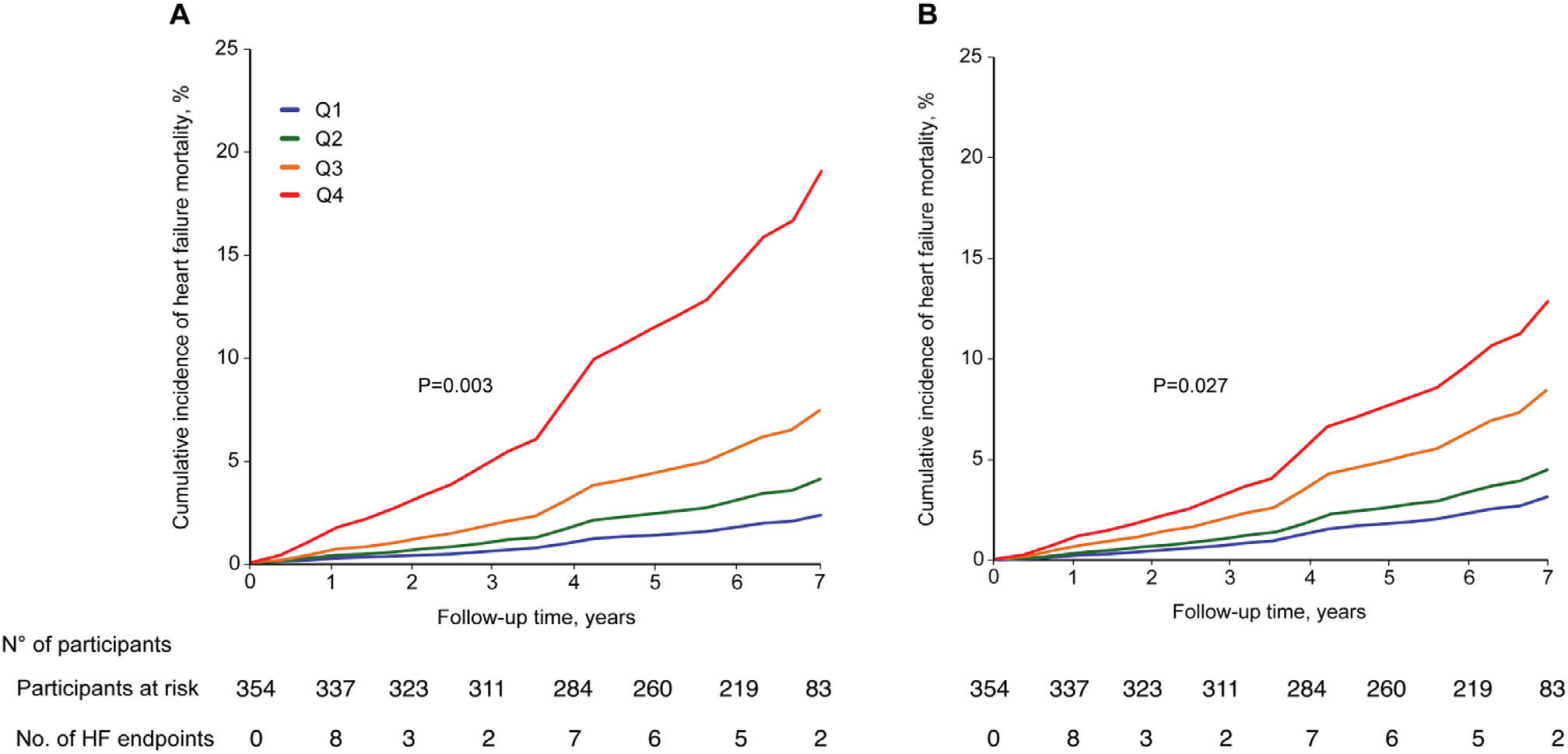
Cumulative incidence of heart failure deaths stratified by the quartiles of (A) urinary classifier scores and (B) serum classifier scores. Among the 354 patients included, 33 heart failure deaths occurred. The cumulative incidence was adjusted for age, sex, body mass index, history of ischemic disease, heart rate, and serum creatinine. Q1 to Q4 represent the highest quartile to the lowest quartile of the classifier score. *P*‐values for trend were derived by Cox proportional hazards regression. The corresponding number of subjects at risk and the number of HF endpoints at yearly intervals are shown below the figure

To the best of knowledge, our study is the first one investigating a large number of collagen I fragments (N = 293) with a focus on collagen degradation. We observed that patients who died from heart failure exhibited (i) specific changes in urinary COL1A1 fragments including an increase in abundance of terminal fragments and, conversely, a decrease in central fragments and (ii) an increase in serum collagen degradation biomarkers, but no significant change in the collagen synthesis biomarker. Both urinary collagen I fragments and serum collagen I degradation biomarkers can independently predict adverse cardiovascular outcomes.

We hypothesize that the observed pattern (Figure 1) is a consequence of differential degradation between termini and the central part of the collagen I molecule. Immediately after procollagen synthesis, collagen I undergoes chemical cross‐linking and modifications. Cross‐linking stabilizes collagen by forming insoluble fibers with increased thickness and stiffness.^5^ Cross‐linking increases over time as a result of chemical modification with aging, inflammation, and cardiomyopathies,^12^ rendering collagen fibers more resistant to degradation.^13^ Since the impact of modification is expected to be more pronounced in the central, rigid part and reduced in the terminal regions (due to increased terminal flexibility), collagen fragments from the central part of COL1A1 are reduced in the group with poor cardiovascular outcomes, indicating reduced degradation of this part of the collagen molecule as a result of increased cross‐linking. This hypothesis also fits with the observed upregulation of MMP‐1: to counteract the attenuation of collagen degradation by cross‐linking, protease activity is upregulated. Study limitations include that we could not test the observation and the classifiers in an independent cohort. Additionally, due to lack of power, we could not account for the multiple testing of 293 fragments or assess the presence of other fibrotic diseases that might affect collagen levels. However, it should be noted that there were no reported signs of fibrotic diseases in the participants.

In conclusion, our results indicate that attenuation of collagen degradation may be responsible for progressive fibrosis in heart failure, with cross‐linking of collagen fibers potentially being a major cause. Urinary markers of collagen I turnover might be a valuable prognostic tool for fibrotic heart disease, possibly enabling the monitoring of treatments targeting fibrosis.

## CONFLICT OF INTEREST

Harald Mischak is the co‐founder and co‐owner of Mosaiques Diagnostics GmbH. Tianlin He and Agnieszka Latosinska are employed by Mosaiques Diagnostics GmbH. The Research Institute Alliance for the Promotion of Preventive Medicine (JAS) received a nonbinding grant from Omron Healthcare Inc. Ltd., Kyoto, Japan. All other authors declared no conflict of interest.

## ETHICS APPROVAL AND CONSENT TO PARTICIPATE

The study complied with the Helsinki declaration for research in humans. Participants provided informed written consent for their data and biological samples to be stored and analyzed. The Ethics Committee of the University of Aachen approved the secondary use of anonymized data and the biomarker measurements (number EK163/19).

## Supporting information

Supporting Information
