# Supplementary material for: Serum and urinary biomarkers of collagen type‐I turnover predict prognosis in patients with heart failure
Source: Clin Transl Med. 2021 Jan 12;11(1):e267. doi: 10.1002/ctm2.267 (PMC7803349; doi:10.1002/ctm2.267)

# Supplementary Data

Supplementary References:

11. Girolami M, Mischak H, Krebs R. Analysis of complex, multidimensional datasets. *Drug Discov Today Technol*. 2006;3(1):13-19. doi:10.1016/j.ddtec.2006.03.010

12. Horn MA, Trafford AW. Aging and the cardiac collagen matrix: Novel mediators of fibrotic remodelling. *J Mol Cell Cardiol*. 2016;93:175-185. doi:10.1016/j.yjmcc.2015.11.005

13. Visse R, Nagase H. Matrix metalloproteinases and tissue inhibitors of metalloproteinases: structure, function, and biochemistry. *Circ Res*. 2003;92(8):827-839. doi:10.1161/01.RES.0000070112.80711.3D

| Table S1 | Baseline characteristics of participants. |
| --- | --- |
| Table S2 | Eight Urinary COL1A1 Fragments Significantly Associated with Heart Failure Death in 354 patients. |
| Table S3 | Components of the urinary and serum classifiers. |
| Table S4 | Cox regression of two classifiers with A) heart failure death, B) cardiovascular death, C) total mortality and D) non-cardiovascular death in 354 patients. There are 33 heart failure deaths, 76 cardiovascular deaths, and 49 non-cardiovascular deaths. |
| Figure S1 | The structure of the collagen I molecule, and the circulating biomarker of its turnover. |
| Figure S2 | Correlation of the abundance of eight significant urinary COL1A1 and three serum biomarkers in 354 patients. |
| Figure S3 | Manhattan plot of peptides associated with heart failure death, cardiovascular death, and non-cardiovascular death. There are 33 heart failure deaths, 76 cardiovascular deaths, and 49 non-cardiovascular deaths. |
|  |  |

# Table S1

**Baseline characteristics of participants.**

| **Baseline Characteristics** | | **All Patients** |  | **Survivors** |  | **HF deaths** | **Non-HF deaths** | **CV deaths** | **Non-CV deaths** |
| --- | --- | --- | --- | --- | --- | --- | --- | --- | --- |
|  |  | **n=354** |  | **n=229** |  | **n=33** | **n=92** | **n=76** | **n=49** |
| Age (years) | | 70.1±10.1 |  | 67.7±10.3 |  | 73.8±8.50 | 74.7±7.93 | 73.8±8.69 | 75.4±6.95 |
| Men | | 257(72.6) |  | 158(69.0) |  | 29(87.9) | 70(76.1) | 63(82.9) | 36(73.5) |
| Body mass index (kg/m^2^) | | 30.0±5.68 |  | 30.5±5.77 |  | 28.6±4.20 | 29.2±5.78 | 28.5±4.92 | 29.9±6.02 |
| Office blood pressure (mmHg)† | |  |  |  |  |  |  |  |  |
| Systolic BP (mmHg) | | 127(112-142) |  | 129(114-143) |  | 112(102-122) | 128(113-143) | 122(108-138) | 125(113-144) |
| Diastolic BP (mmHg) | | 71(62-80) |  | 72(65-81) |  | 64(59-70) | 71(61-79) | 67(60-77) | 70(61-79) |
| Mean arterial pressure (mmHg) | | 91(80-101) |  | 92(82-101) |  | 80(77-84) | 90(79-99) | 83(77-97) | 90(79-100) |
| Heart rate (beats per minute) | | 65(59-74) |  | 64(59-73) |  | 69(60-76) | 66(60-73) | 67(60-76) | 65(60-72) |
| Co-morbidities | |  |  |  |  |  |  |  |  |
| Hypertension‡ | | 337(95.2) |  | 213(93.0) |  | 32(97.0) | 92(100) | 75(98.7) | 49(100) |
| Diabetes mellitus§ | | 129(36.4) |  | 81(35.4) |  | 8(24.2) | 40(43.5) | 29(38.2) | 19(38.8) |
| Current smokers | | 235(66.4) |  | 153(66.8) |  | 22(66.7) | 60(65.2) | 48(63.2) | 34(69.4) |
| Drinking alcohol | | 1167(47.2) |  | 116(50.7) |  | 17(51.5) | 33(35.9) | 28(36.8) | 22(44.9) |
| Ischemic heart disease | | 200(56.5) |  | 108(47.2) |  | 29(87.9) | 63(68.5) | 59(77.6) | 33(67.3) |
| Heart failure | |  |  |  |  |  |  |  |  |
| Atrial fibrillation | | 86(24.3) |  | 51(22.3) |  | 6(18.2) | 29(31.5) | 19(25.0) | 16(32.7) |
| HFrEF (LVEF<40%) | | 132(37.3) |  | 70(30.6) |  | 23(69.7) | 39(42.4) | 43(56.6) | 19(38.8) |
| HFmrEF (LVEF 40->50%) | | 70(19.8) |  | 47(20.5) |  | 5(15.2) | 18(19.6) | 13(17.1) | 10(20.4) |
| HFpEF (LVEF>=50%) | | 152(42.9) |  | 112(48.9) |  | 5(15.2) | 35(38.0) | 20(26.3) | 20(40.8) |
| Use of medications | |  |  |  |  |  |  |  |  |
| ACEs/ARB | | 223(63.0) |  | 135(59.0) |  | 23(69.7) | 65(70.7) | 54(71.1) | 34(69.4) |
| Diuretics | | 188(53.1) |  | 93(40.6) |  | 29(87.9) | 66(71.7) | 62(81.6) | 33(67.3) |
| MRA | | 164(46.3) |  | 89(38.9) |  | 26(78.8) | 49(53.3) | 50(65.8) | 25(51.0) |
| Beta-blockers | | 280(79.1) |  | 174(76.0) |  | 31(93.9) | 75(81.5) | 64(84.2) | 42(85.7) |
| Biochemical data | |  |  |  |  |  |  |  |  |
| Serum creatinine (mmol/L) | | 98.0(81.0-126) |  | 92(75-120) |  | 118(106-160) | 108(90-155) | 111(94.8-160) | 111(89-154) |
| Hemoglobin (g/dL) | | 13.5(12.1-14.5) |  | 13.6(12.3-14.6) |  | 13.4(11.6-14.2) | 13.3(11.7-14.2) | 12.8±1.73 | 13.4(14.0) |
| Plasma NT-proBNP (pg/mL) | | 624(253-1550) |  | 507(197-1120) |  | 2260(1670-3120) | 1120(542-3040) | 2003(786-3500) | 956(393-2460) |
| Serum collagen biomarkers | |  |  |  |  |  |  |  |  |
| PICP (ng/mL) | | 84.6(69.6-107) |  | 84.7(70.6-104) |  | 85.7(64.3-111) | 83.4(69.8-112) | 84.8(68.5-123) | 84.4(67.7-123) |
| CITP (ng/mL) | | 3.65(2.87-5.10) |  | 3.36(2.65-4.39) |  | 4.26(3.32-7.41) | 4.20(3.39-6.58) | 4.19(3.36-6.78) | 4.27(3.33-6.54) |
| MMP-1 (pg/mL) | | 11.3(6.90-16.4) |  | 10.3(6.47-15.7) |  | 13.5(9.36-19.6) | 13.1(7.58-16.7) | 12.3(8.85-19.2) | 13.7(7.29-16.4) |
| CITP: MMP-1 | 372(218-566) | |  | 367(212-523) |  | 361(232-553) | 411(227-681) | 365(224-556) | 438(256-698) |
| PICP: CITP | 22.5(16.5-31.7) | |  | 25.4(17.6-33.8) |  | 19.0(14.7-22.9) | 19.5(14.3-27.5) | 19.1(14.6-25.8) | 18.8(14.1-26.6) |

Values are given as n(%), or median (interquartile range), or mean ± standard deviation. †Office blood pressure was the average of five consecutive readings. ‡Hypertension was an office blood pressure of ≥140 mmHg systolic, or ≥90 mm Hg diastolic or the use of antihypertensive drugs. §Diabetes Mellitus was a self-reported diagnosis.

HF = heart failure, CV = cardiovascular; BP = blood pressure, HFmrEF = heart failure with mid-range ejection fraction, HFpEF = heart failure with preserved ejection fraction, HFrEF = heart failure with reduced ejection fraction, PICP = carboxy-terminal propeptide of procollagen type I, CITP = carboxy-terminal telopeptide of collagen type I, MMP-1 = matrix metalloproteinase 1, NT-proBNP = N-terminal pro-B type natriuretic peptide.

# **Table S2**

**Eight Urinary COL1A1 Fragments Significantly Associated with Heart Failure Death in 354 patients.**

| **ID** | **Start Position** | **Sequence** | **HR (95% CI)** | **p-value** |
| --- | --- | --- | --- | --- |
| e10277 | 229 | nGDDGEAGKpGRPGERGPPGp | 1.53 (1.01-2.30) | 0.043 |
| e00214 | 522 | SpGEAGRpG | 0.60 (0.37-0.96) | 0.035 |
| e06441 | 541 | TGSpGSpGPDGKTGPpGP | 0.62 (0.41-0.93) | 0.023 |
| e05560 | 542 | GSpGSpGPDGKTGPPGp | 0.56 (0.37-0.84) | 0.005 |
| e01399 | 562 | DGRpGPpGPpG | 0.55 (0.31-0.98) | 0.042 |
| e05671 | 657 | KpGEQGVpGDLGAPGp | 0.68 (0.46-0.99) | 0.043 |
| e08489 | 1023 | SpGRDGSpGAKGDRGETGP | 1.51 (1.02-2.22) | 0.036 |
| e05329 | 1178 | GPpGPpGPpGPPGPPSA | 1.74 (1.20-2.54) | 0.003 |

HR indicates the relative risk associated with a unitary increase in the rank-normalized fragment abundance in a multivariable Cox regression model. The model was adjusted for age, sex, body mass index, history of ischemic disease, heart rate, and serum creatinine**.** Fragments that were positively associated with HF death are colored in red, and those negatively associated with HF death are colored in blue. The fragments are ordered by position from the N-terminal of COL1A1. HR = hazard ratio, CI = confidence interval of the hazard ratio.

**Table S3**

**Components of the urinary and serum classifiers**

| Classifier | C | Gamma | Components |
| --- | --- | --- | --- |
| Urinary classifier | 40960 | 0.0003200 | 8 urinary COL1A1 fragments:   \| e00214(SpGEAGRpG), \| \| --- \| \| e01399(DGRpGPpGPpG), \| \| e05329(GPpGPpGPpGPPGPPSA), \| \| e05560(GSpGSpGPDGKTGPPGp), \| \| e05671(KpGEQGVpGDLGAPGp), \| \| e06441(TGSpGSpGPDGKTGPpGP), \| \| e08489(SpGRDGSpGAKGDRGETGP), \| \| e10277(nGDDGEAGKpGRPGERGPPGp), \| |
| Serum classifier | 256.0 | 0.2048 | 3 serum biomarkers: CITP, MMP-1, PICP |

C and Gamma are parameters obtained from SVM modelling. CITP = carboxy-terminal telopeptide of type I collagen, MMP-1 = matrix metalloproteinase 1; PICP = procollagen type I carboxy-terminal propeptide.

**Table S4**

**Cox regression of two classifiers with A) heart failure death, B) cardiovascular death, C) total mortality and D) non-cardiovascular death in 354 patients. There are 33 heart failure deaths, 76 cardiovascular deaths, and 49 non-cardiovascular deaths.**

|  | **Univariable Model** | | **Multivariable Model 1**† | | **Multivariable Model 2**‡ | |
| --- | --- | --- | --- | --- | --- | --- |
|  | **HR (95% CI)** | **p-value** | **HR (95% CI)** | **p-value** | **HR (95% CI)** | **p-value** |
| A) Heart failure death |  |  |  |  |  |  |
| Urinary classifier | 2.17 (1.57-2.98) | <0.0001 | 1.86 (1.34-3.80) | <0.001 | 1.58 (1.13-3.11) | 0.007 |
| Serum classifier | 2.20 (1.46-3.32) | <0.001 | 1.74 (1.11-3.03) | 0.017 | 1.59 (1.01-2.75) | 0.044 |
| B) Cardiovascular death |  |  |  |  |  |  |
| Urinary classifier | 1.80 (1.45-2.23) | <0.0001 | 1.58 (1.26-1.98) | <0.0001 | 1.36 (1.09-2.96) | 0.008 |
| Serum classifier | 1.94 (1.48-2.56) | <0.0001 | 1.65 (1.22-2.22) | <0.001 | 1.44 (1.06-2.87) | 0.022 |
| C) Total mortality |  |  |  |  |  |  |
| Urinary classifier | 1.64 (1.38-1.95) | <0.0001 | 1.45 (1.21-3.36) | <0.0001 | 1.30 (1.08-2.95) | 0.005 |
| Serum classifier | 1.90 (1.53-2.36) | <0.0001 | 1.68 (1.33-3.77) | <0.0001 | 1.45 (1.12-3.07) | 0.005 |
| D) Non-cardiovascular death |  |  |  |  |  |  |
| Urinary classifier | 1.50 (1.13-1.99) | 0.006 | 1.32 (0.94-1.67) | 0.058 | 1.25 (0.911-2.49) | 0.165 |
| Serum classifier | 1.92 (1.35-2.72) | <0.001 | 1.78 (1.18-2.47) | 0.002 | 1.51 (0.962-2.37) | 0.077 |

HR = hazard ratio, CI = confidence interval of the hazard ratio.

Univariable Model: unadjusted.

†Multivariable Model 1: urinary classifier and serum classifier were included as covariables.

‡Multivariable Model 2: adjusted for age, sex, body mass index, history of ischemic heart disease, heart rate, serum creatinine, and NT-proBNP.

# Figure S1

**The structure of the collagen I molecule, and the circulating biomarker of its turnover.** The triple alpha-helix structure of the collagen molecule, which is composed of two alpha-1 chains and one alpha-2 chain. The two pro-peptide domains are cleaved during collagen synthesis, forming a mature collagen molecule. Collagen degradation by MMPs results in the release of telopeptides into plasma; MMPs are inhibited by TIMP-1. CITP = carboxy-terminal telopeptide of type I collagen, MMPs = matrix metalloproteinases, PICP = procollagen type I carboxy-terminal propeptide, TIMP-1 = tissue inhibitor of matrix metalloproteinases


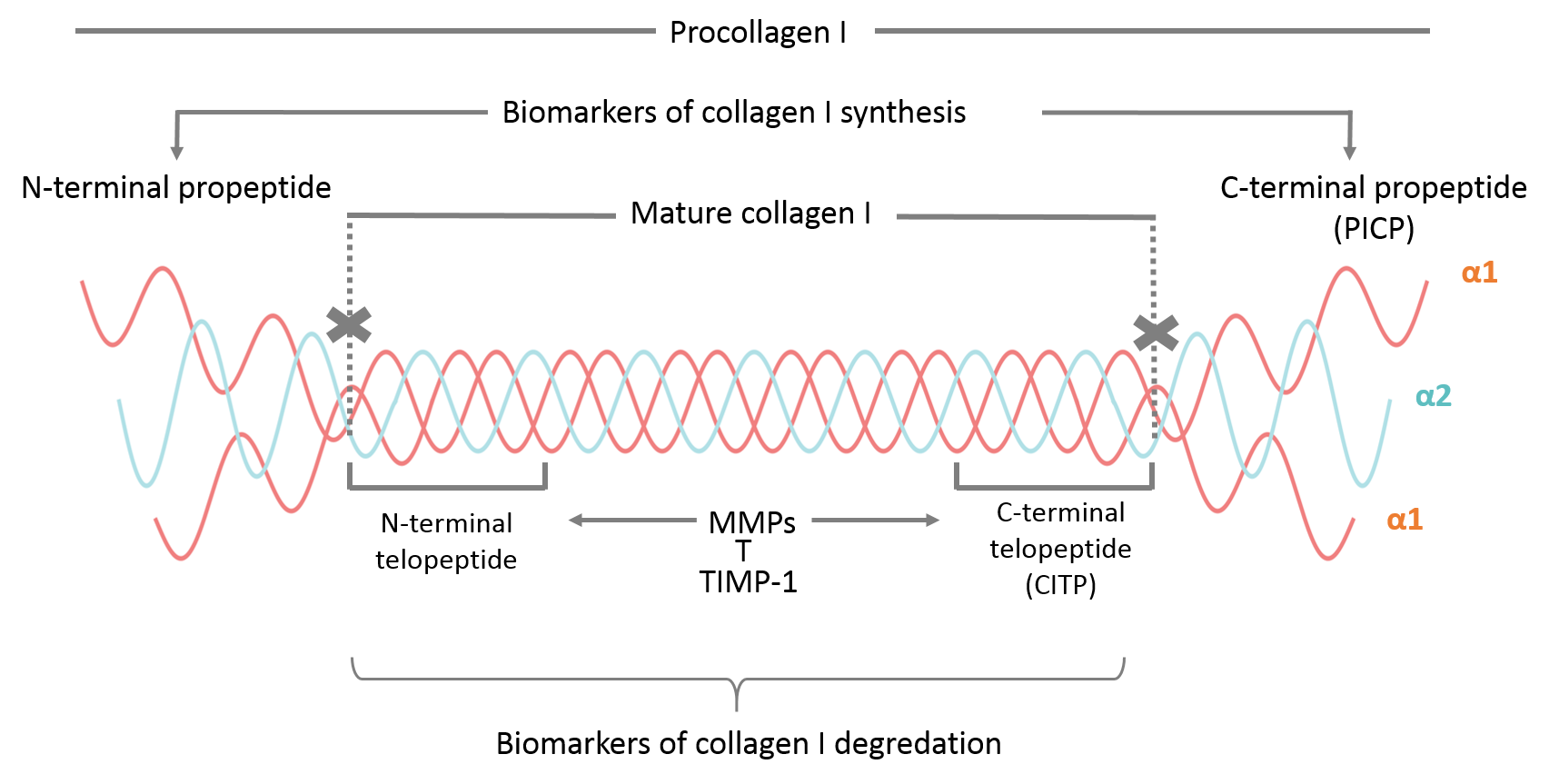


# Figure S2

Correlation of the abundance of eight significant urinary COL1A1 and three serum biomarkers in 354 patients. The correlation was calculated with Spearman‘s test. CITP = carboxy-terminal telopeptide of type I collagen, MMP-1 = matrix metalloproteinase 1, PICP = procollagen type I carboxy-terminal propeptide.


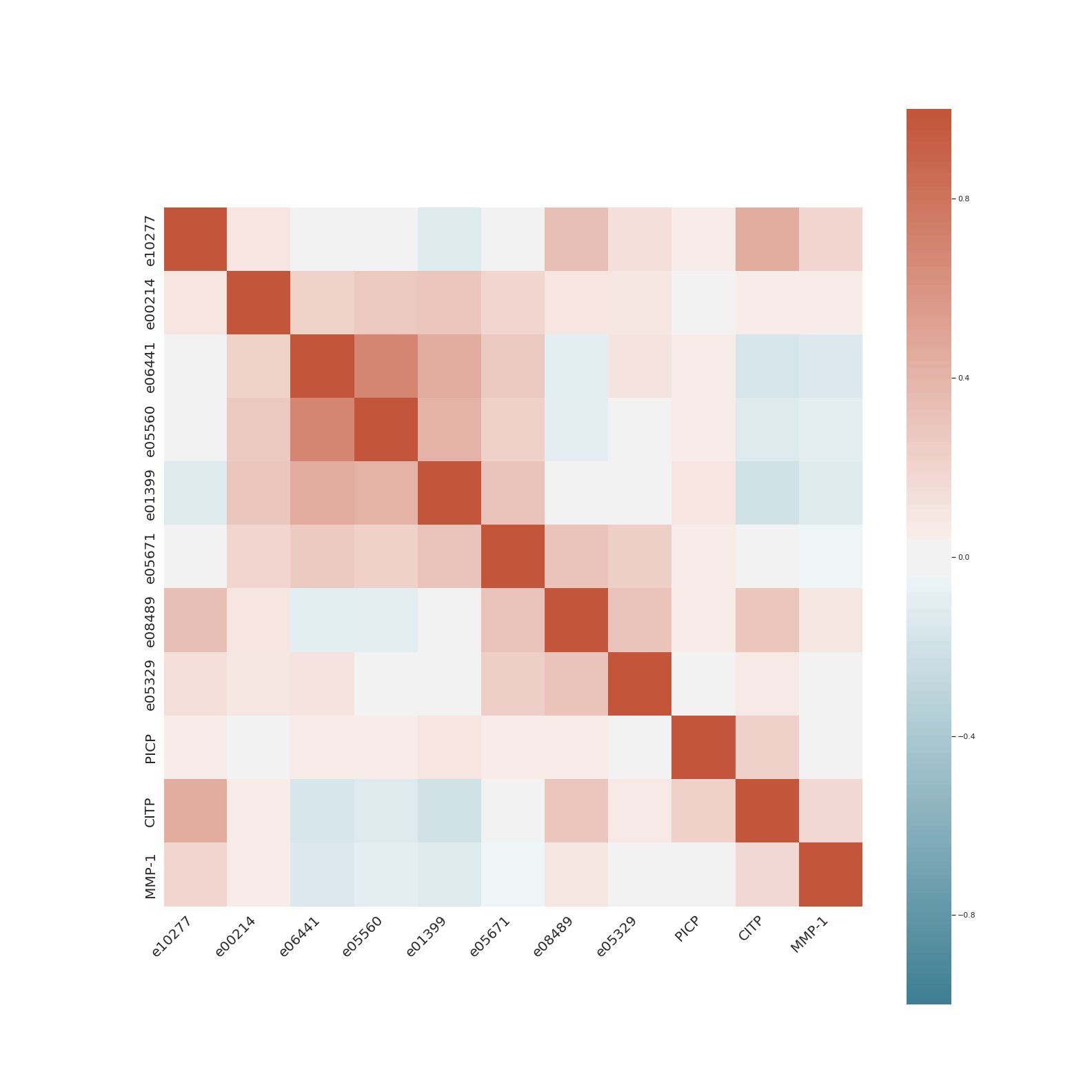


# Figure S3

**Manhattan plot of urinary COL1A1 fragments associated with heart failure death, cardiovascular death, and non-cardiovascular death.** There are 33 heart failure deaths, 76 cardiovascular deaths, and 49 non-cardiovascular deaths. The -log10(P) probability of urinary COL1A1 fragments in a multivariable proportional hazard model. The model was adjusted for age, sex, body mass index, history of ischemic disease, heart rate, and serum creatinine. The six panels (from left to right) show the fragments that were associated with 1) increase in heart failure death, 2) decrease in heart failure death, 3) increase in CV death, 4) decrease in CV death, 5) increase in non-CV death and 6) decrease in non-CV death. The two horizontal lines denote the significance thresholds at p=0.01 and p=0.05. Only the fragments with a p<0.05 were annotated. The fragments significantly associated (either positively or negatively) with both HF death and CV death were highlighted. CV = cardiovascular, HF = heart failure.


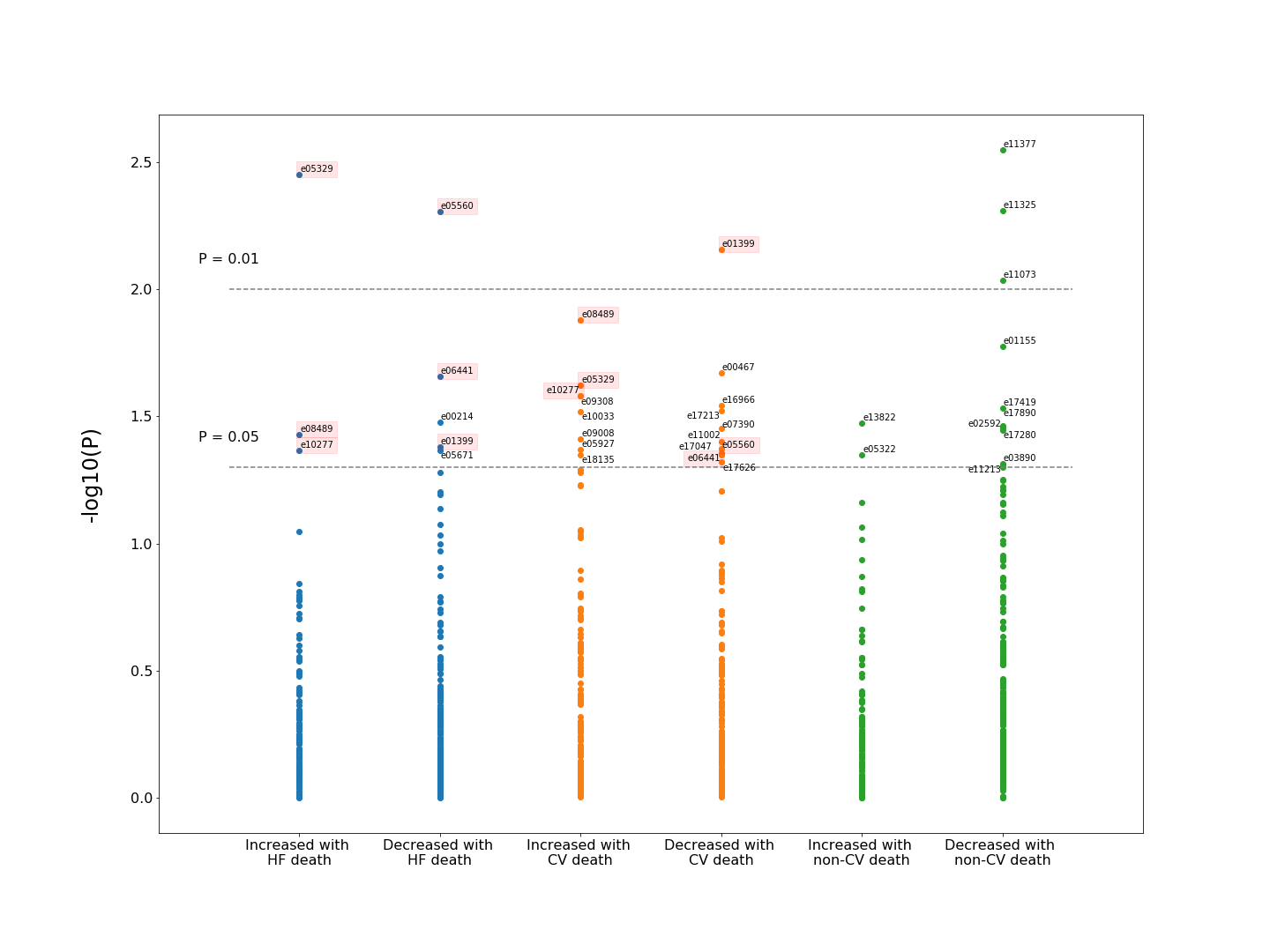

Supplement: Supplementary file 1 — Supporting Information [file CTM2-11-e267-s001.docx]
